# Impact of Tumour Epstein–Barr Virus Status on Clinical Outcome in Patients with Classical Hodgkin Lymphoma (cHL): A Review of the Literature and Analysis of a Clinical Trial Cohort of Children with cHL

**DOI:** 10.3390/cancers14174297

**Published:** 2022-09-01

**Authors:** Mahdi Nohtani, Katerina Vrzalikova, Maha Ibrahim, Judith E. Powell, Éanna Fennell, Susan Morgan, Richard Grundy, Keith McCarthy, Sarah Dewberry, Jan Bouchal, Katerina Bouchalova, Pamela Kearns, Paul G. Murray

**Affiliations:** 1 Bernal Institute, University of Limerick, V94 T9PX Limerick, Ireland; 2 School of Medicine, University of Limerick, V94 T9PX Limerick, Ireland; 3 Institute of Immunology and Immunotherapy, University of Birmingham, Edgbaston, Birmingham B15 2TT, UK; 4 Cancer Pathology Department, South Egypt Cancer Institute, Assiut University, Assiut 71526, Egypt; 5 Department of Public Health & Epidemiology, University of Birmingham, Birmingham B15 2TT, UK; 6 Department of Histopathology, Sheffield Teaching Hospitals, Sheffield S10 2JF, UK; 7 Medical School, Queen’s Medical Centre, Nottingham NG7 2UH, UK; 8 Department of Histopathology, Wye Valley NHS Trust, Hereford HR1 2ER, UK; 9 Cancer Research UK Clinical Trials Unit (CRCTU), Institute of Cancer and Genomic Sciences, College of Medicine and Dental Sciences, University of Birmingham, Edgbaston, Birmingham B15 2TT, UK; 10 Department of Clinical and Molecular Pathology, Institute of Molecular and Translational Medicine, Palacky University, 77900 Olomouc, Czech Republic; 11 Department of Pediatrics, Faculty of Medicine and Dentistry, Palacky University, 77900 Olomouc, Czech Republic

**Keywords:** classic Hodgkin lymphoma (cHL), Epstein–Barr virus (EBV), clinical trial

## Abstract

**Simple Summary:**

The Epstein–Barr virus (EBV) contributes to different forms of human cancer, including a subset of classical Hodgkin lymphoma (cHL), a B-cell lymphoma with unusual histological features. Although the pathogenesis of EBV-associated cHL remains to be elucidated, biological investigations point to an important aetiological role for the virus in the development of this tumour. This is even more relevant now considering the potential opportunities that exist to treat EBV-associated disorders, for example, with immunotherapeutics or small molecule inhibitors targeting viral proteins. For this reason, we believe it is now timely to review the association between EBV and cHL and in particular to re-evaluate the impact of EBV status on clinical outcomes in cHL patients. Herein, we also report the impact of EBV on clinical outcomes in a cohort of children and adolescents with cHL.

**Abstract:**

In this study, we have re-evaluated how EBV status influences clinical outcome. To accomplish this, we performed a literature review of all studies that have reported the effect of EBV status on patient outcome and also explored the effect of EBV positivity on outcome in a clinical trial of children with cHL from the UK. Our literature review revealed that almost all studies of older adults/elderly patients have reported an adverse effect of an EBV-positive status on outcome. In younger adults with cHL, EBV-positive status was either associated with a moderate beneficial effect or no effect, and the results in children and adolescents were conflicting. Our own analysis of a series of 166 children with cHL revealed no difference in overall survival between EBV-positive and EBV-negative groups (*p* = 0.942, log rank test). However, EBV-positive subjects had significantly longer event-free survival (*p* = 0.0026). Positive latent membrane protein 1 (LMP1) status was associated with a significantly lower risk of treatment failure in a Cox regression model (HR = 0.21, *p* = 0.005). In models that controlled for age, gender, and stage, EBV status had a similar effect size and statistical significance. This study highlights the age-related impact of EBV status on outcome in cHL patients and suggests different pathogenic effects of EBV at different stages of life.

## 1. Introduction

Classical Hodgkin lymphoma (cHL) is characterised by rare malignant Hodgkin/Reed Sternberg (HRS) cells surrounded by a florid tumour microenvironment (TME) comprising different inflammatory cells. HRS cells are germinal centre (GC)-experienced B cells [1] but do not express a functional B-cell receptor (BCR); survival of these cells is mediated by activation of anti-apoptosis pathways crucial for cHL pathogenesis [2]. 

Following B-cell infection with EBV, extrachromosomal copies of circular genomes, known as episomes, are created by fusion of the viral terminal repeats (TRs) creating termini of a unique length [3]. The detection of TRs of different lengths indicates more than one infection event [3]. In contrast, TRs with an identical number of repeats are found in cHL and other EBV-associated cancers, indicating expansion from a single infected cell [4]. EBV is crucial also for cHL progression, as it is found during the course of cHL [5].

EBV protein expression in HRS cells is limited to (i) Epstein–Barr virus nuclear antigen-1 (EBNA1) [6], an essential replication and virus maintenance factor, as well as a transcription factor that regulates the expression of both viral and cellular genes [7,8,9,10]; EBNA1 has been shown to promote the growth and survival of cHL-derived cell lines [11,12] and (ii) two latent membrane proteins (LMPs) [13,14,15]; LMP1 is a constitutively active CD40 receptor [16] that activates oncogenic pathways including NF-κB, JAK/STAT, AP-1, and phospatidylinositol-3 kinase (PI3K)/AKT [17,18,19,20], whereas LMP2A is a BCR homologue [21,22] that can promote the survival of BCR-negative B cells [23,24,25].

Serological studies showing that antibody levels to EBV antigens were raised in HL patients provided early evidence for an aetiological role [26,27]. Later, people who develop EBV-positive cHL were shown to be more likely to have elevated antibody levels to EBV antigens compared to those who develop EBV-negative cHL [28]. Furthermore, infectious mononucleosis (IM), representing symptomatic primary EBV infection, was shown to increase the risk of EBV-positive, but not EBV-negative, cHL [29,30,31,32], and a seasonal peak in children diagnosed with cHL was shown for EBV-positive, but not for EBV-negative, cHL [33]; these data point to primary EBV infection as a trigger for the development of EBV-positive cHL. While only a small fraction of people infected with EBV will develop EBV-positive cHL [34], susceptibility is associated with variation in the human leukocyte antigen (HLA) region [35,36,37]; those with HLA-A*01 have an increased risk, and those with HLA-A*02 have a decreased risk [36,38,39].

EBV is present in HRS cells more frequently in male patients, in those with mixed cellularity disease, and in patients from resource-poor countries. Geographical differences might be explained by differing susceptibilities between ethnic groups [40,41]. For example, EBV-positive cHL is more common in Asians and Hispanics compared with whites or blacks and in South Asian children compared with non-South Asian children in the UK [40,42]. In resource-rich nations, the proportion of cHL with EBV is higher in older people and in children, with lower rates in young adults [43]. Jarrett et al. have proposed a four-disease model: (1) childhood cHL, which is frequently EBV-positive; (2) cHL of older adults, which is also often EBV-related; it is well known that aging is associated with decreased immune function, so this entity may be related to EBV reactivation; (3) EBV-negative disease in young adults, usually, but not always, of nodular sclerosis type; (4) EBV-associated cHL occurring after late EBV exposure [44]. 

While first-line combination chemotherapy and radiotherapy is effective for the majority of cHL patients, prognosis for patients with refractory or relapsed disease remains dismal, despite the use of newer targeted therapies such as brentuximab vedotin. The current treatments also cause significant long-term toxicities that include secondary malignancies, cardiopulmonary toxicity, hypothyroidism, and infertility. Outcomes are particularly poor for older patients (5-year survival 30–50% in patients older than 70 years). Despite overwhelming evidence supporting an aetiological role for EBV in cHL, EBV status does not currently influence patient management. Moreover, therapies designed to specifically target EBV are yet to be adopted. 

In this study, we have performed a comprehensive literature review encompassing all relevant previous studies reporting the effect of EBV status on patient outcome. We have also separately explored the effect of EBV positivity in a clinical trial of children and adolescents with cHL from the United Kingdom in an attempt to clarify the apparently conflicting effects of EBV status on clinical outcomes in the paediatric setting. 

## 2. Materials and Methods

### 2.1. Literature Review

Using the keywords “EBV” and “Hodgkin lymphoma,” we conducted a search on PubMed (http://www.ncbi.nlm.nih.gov/pubmed; accessed on 2 February 2022). Additionally, we looked through the reference lists of the papers and manually included the publications missed by the original search. Using the title, names and affiliations of the authors, duplicate information or overlapping articles were eliminated. We confined our analysis to studies that used either in situ hybridisation to detect the Epstein–Barr-encoded RNAs (EBER1 and EBER2) and/or immunohistochemistry to identify expression of the EBV oncogene, LMP1. We excluded studies in which other virus proteins (LMP2A and EBNA1, which are less abundant in EBV-positive HRS cells) were used as targets. We also excluded studies that exclusively used PCR to detect EBV. NLPHL is regarded as a distinct disease entity, and it differs from cHL primarily in terms of morphology, phenotype, genetics, clinical behaviour and EBV positivity. Therefore, inclusion of these cases in studies was taken into account.

### 2.2. Analysis of a Clinical Trial Cohort of Children and Adolescents with cHL

#### Patients

Eligible patients included all children and adolescents (<18 years old) with newly diagnosed, untreated, biopsy-proven classical (i.e., excluding lymphocyte predominant subtype) HL who had been enrolled onto the HD 2000 02 (HD 3 trial) and for whom archival pathological material was available as FFPE sections (*n* = 189). Ethical approval and written informed consent were obtained from all patients and/or their parents/guardians in accordance with the then-current institutional and ethical committee guidelines. The results of this clinical trial and details of patient recruitment and treatment regimens have been reported previously [45].

### 2.3. Event-Free Survival and Overall Survival

Event-free survival (EFS) was calculated as the time from the date of diagnosis to the date of relapse, progression, or death from any cause. Patients who did not experience any events were censored at their last follow-up visit. Overall survival (OS) was measured from the date of diagnosis to the date of last follow-up visit or to the date of death.

### 2.4. EBV Detection

Immunohistochemistry for LMP1 was performed on paraffin sections from each case to detect the presence of EBV infection as previously described [14]. Specimens were recorded as either EBV-positive (LMP1 present within HRS cells) or EBV-negative (LMP1 not detectable in HRS cells). 

### 2.5. Statistical Methods

Mann–Whitney U- and chi-squared tests were used to detect differences between EBV-positive and EBV-negative groups in terms of patient and disease characteristics. Differences in survival and event-free survival between EBV-positive and EBV-negative patients were investigated using Kaplan–Meier curves and log-rank tests. A Cox proportional hazards univariate analysis was also performed to ascertain the hazard ratio (HR) for event-free survival of each variable. The variables considered were EBV, age (≤12 and >12), sex, and disease stage early stages I/IIA and advanced stages IIB/III/IV). Multivariate Cox analysis was performed to determine which factors were independently predictive of event-free survival. Life-table methods were used to derive treatment failure rates (hazard) and the hazard ratio by EBV status at various time intervals after diagnosis. In order to determine that the 189patients that were eventually used in our analysis were representative of the whole cohort, chi-squared and *t*-tests were used to compare age, gender, subtype, stage, and symptoms between the two groups. All analyses used either SPSS Version 16 (SPSS Inc., Chicago, IL, USA) or R-4.1.2, and differences were deemed significant if the *p*-value was less than 0.05.

## 3. Results

### 3.1. Literature Review

A total of 40 studies met the inclusion criteria set out above (Supplementary Table S1). We focused our analysis on those studies that had categorized patients by age group. We summarize the results of our analysis below.

*Older adults/elderly patients with cHL:* our literature review revealed that an EBV-positive status was associated with poor prognosis in older adults/elderly patients in six of seven studies [46,47,48,49,50,51,52]. 

*Young adults with cHL:* A modest beneficial effect of an EBV-positive status for young adults with cHL was demonstrated in four of eight studies. Three studies showed no significant effect of EBV, and one showed a negative impact of EBV on prognosis [46,47,48,53,54,55,56,57].

*Paediatric and adolescent cHL:* Eight studies explored the effect of EBV status on outcome in children or adolescents with cHL (Table 1). Engel et al. studied 47 patients (with follow up available on 36) and found significantly fewer deaths and longer median survival in EBV-positive cases [58]. Barros et al. studied 104 patients and showed that EBV was significantly associated with lower-risk nodular sclerosis disease [59]. Keegan et al. showed that in children <15 years old, EBV positivity was associated with longer survival, but this was only of borderline significance [46]. Three studies reported no significant effect of EBV status on outcome [60,61,62]. Two studies reported a negative effect of EBV on outcome in children. In the largest study to date, Claviez [63] reported the impact of EBV status on outcome in 842 children and adolescents. They found that LMP1 positivity was associated with significantly poorer overall survival (OS) but found no effect on failure-free survival (FFS); in fact, in their study, FFS was higher in EBV-positive (89.1%) compared with EBV-negative (84.1%) patients. In the second study, Koh et al. reported that in 135 children with HL, EBER positivity was associated with a significantly higher international prognostic score (IPS) and significantly lower OS, although positivity was not an independent risk factor for OS [64]. The authors did not report the influence of EBV status on FFS.

### 3.2. Analysis of a Clinical Trial Cohort of Children and Adolescents with cHL

Given the observed uncertainty of EBV’s effects on outcome in childhood and adolescent HL, we next examined the effects of EBV status on EFS and OS in a cohort of children and adolescents with cHL recruited to a clinical trial in the United Kingdom, where full clinical annotation of the sample collection, including reliable treatment, follow up, and outcome data were available. This trial has been reported previously, but EBV status has not been examined in this cohort before [45].

EBV status was tested in tissue from 189 trial subjects. Of these samples, 23 were excluded (six—identity uncertain; five—not lymphoid tissue; seven—test failed; one—repeated tests contradictory; four—not HL at Pathology Review). Sixty-two of the remaining 166 cases were EBV positive (34.4%, 95% CU 27.6–41.2%). The 189 patients included in this study were representative of the whole clinical trial cohort of 387 patients (Table 2).

Characteristics of the subjects, according to EBV status, are shown in Table 3. EBV-positive patients were significantly younger than EBV-negative patients, with a median age of 10.0 years, compared to 14.2 for EBV-negative subjects. EBV-positive cases were also less likely to have stage IV disease. The subtypes also differed significantly, EBV-positive tumours being less likely to be of nodular sclerosis subtype and more likely to be of mixed cellularity subtype.

**Figure 1:**
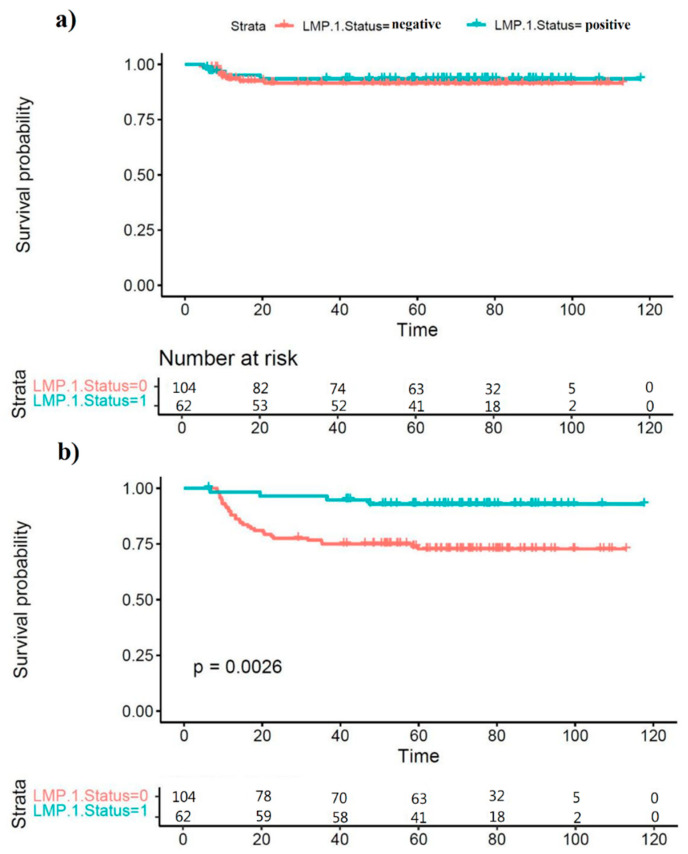
Overall survival (**a**) and event-free survival (**b**) for 166 subjects with cHL, by EBV status.

In total, 14 deaths and 47 events were recorded (relapses, disease progression, persistent disease). Figure 1 shows Kaplan–Meier survival curves for overall survival (Figure 1a) and event-free survival (Figure 1b), by EBV status. There was no evidence of a difference between groups for overall survival (*p* = 0.942, log rank test). Event-free survival, however, was significantly poorer in EBV-negative subjects (*p* = 0.0026). As described above, fewer EBV-positive patients presented with stage IV disease than patients with EBV-negative cHL. The treatment for patients in this trial varied according to stage: patients with stage II and stage III disease received three cycles of ChlVbPP/ABVcD chemotherapy, whilst those with stage IV disease received four cycles. Therefore, the improved EFS observed for EBV-positive patients is not likely to be a consequence of more intensive chemotherapy.

**Figure 2:**
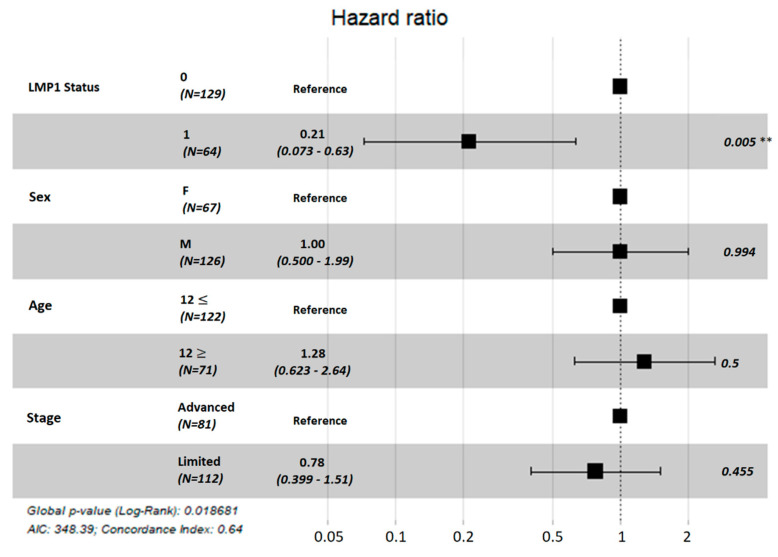
Multivariate analysis of the effect of EBV positivity on event-free survival in 166 cHL patients, adjusting for sex, age, and stage. ** marks p-values less than 0.01.

In a Cox regression model, positive LMP1 status was associated with a significantly reduced risk of treatment failure (HR = 0.21, *p* = 0.005, Figure 2). EBV status retained a similar effect size and statistical significance in models adjusting for age, sex, and stage. We defined stage in two groups, early (stages I/IIA) and advanced (IIB/III/IV), based on risk stratification and treatment management guidelines [86,87]. Patients within the early group had reduced risk of failure compared to advanced stages, but this difference was not statistically significant (*p* = 0.45), and EBV status remained the most predictive factor. Age also has a negative effect on patients’ outcome, with older patients having poorer outcomes, but this was not statistically significant.

## 4. Discussion

Notwithstanding some of the difficulties in drawing comparisons across different studies included in our literature review (different methodologies, patient recruitment, inclusion of NLPHL in some studies, different age cut-offs, etc.), we can draw several general conclusions. Thus, almost all studies of older adults/elderly patients reported an adverse effect of an EBV-positive status on outcome. In contrast, in younger adults with cHL, EBV-positive status was either associated with a moderate beneficial effect or no effect. It is possible that within the older adult group, EBV is not itself a driver of poorer outcomes but might be associated with other co-morbidities including reduced immunity and/or generally poorer health. Nevertheless, it should be noted that many older patients with cHL are unfit to receive standard-of-care combination chemotherapy; therefore, older people with EBV-positive cHL might benefit from therapies that specifically target EBV if they prove to be better tolerated than conventional chemotherapy. For a more detailed discussion of targeting EBV therapeutically in EBV-positive cHL and other cancers, the reader is referred to recent reviews [88,89,90].

The situation at first sight would appear to be less clear in children and adolescents. Thus, we found that of the eight studies that reported an effect of EBV on outcome, three showed a beneficial effect, three no effect, and two an adverse effect. However, it must be pointed out that of the three studies showing no effect, two of these, one from India [60] and the other from Turkey [61], showed very high rates of EBV detection (96.6% and 82.5%, respectively), meaning they were likely insufficiently powered to detect differences even if present. Moreover, of the two studies showing an adverse effect of EBV, one showed poorer overall survival (OS) only in some subgroups of the disease but no effect on failure-free survival (FFS) [63]. The other study found a significantly shorter OS for EBV-positive patients but did not report FFS [64]. It has been suggested previously that OS may not be the best end-point since factors other than those related to the primary treatment may influence outcome [66].

Given the uncertainty of EBV’s effects in the paediatric setting, we decided to study a separate cohort of children and adolescents with cHL recruited to a clinical trial [45]. We found that among 166 patients, EBV had no effect on OS but was associated with a significantly longer FFS. In a Cox regression model, EBV positivity was found to be associated with a significantly reduced risk of treatment failure. Moreover, EBV status retained a similar effect size and statistical significance in models adjusting for age, sex, and stage. Thus, our new data suggest that EBV might be associated with a better prognosis in children and adolescents, at least in the UK, and that it should be considered as a factor that could help stratify patients, for example, for de-escalation of therapy. Given the overall success of conventional therapies in children, OS may not be the best measure of outcome when assessing biological factors such as EBV status and which may be confounded by disease-unrelated deaths.

**Figure 3:**
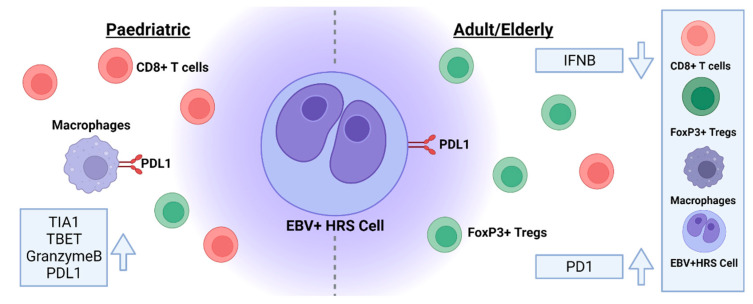
Schematic representation of differences in the cHL TME in different age groups. EBV-positive paediatric cHL has a more cytotoxic TME with predominant Th1 polarisation, overexpression of CD8, TIA1, TBET, and granzyme B, and fewer FoxP3+ regulatory T cells (Tregs) compared with EBV-negative disease. Fewer granzyme B-positive T cells, increased Tregs, and limited interferon beta production in older adult/elderly EBV-positive cHL indicate a more immunosuppressive TME.

The cHL TME has been shown to vary with age and with EBV status (Figure 1). Thus, cytotoxic markers on T cells and numbers of CD16+ natural killer cells are increased in EBV-positive vs. EBV-negative cHL (cases included age-matched paediatric, adult, and elderly patients) [91]. EBV-positive paediatric cHL also exhibits a more cytotoxic TME with predominant Th1 polarisation, overexpression of CD8, TIA1, TBET, and granzyme B, and reduced FoxP3+ regulatory T cells (Tregs) compared with EBV-negative disease (Th2 and Th17) [92,93]. However, increased PDL1+ cells in EBV-positive paediatric cHL might blunt T-cell-mediated cytotoxicity [94]. In adult/elderly EBV-positive cHL, reduced granzyme B-positive T cells, increased Tregs, and limited interferon beta production indicate a more immunosuppressive TME, potentially contributing to the unfavourable outcome found in elderly cHL patients [95,96]. Furthermore, PDL1 is expressed more frequently on EBV-positive HRS cells rather than in the TME, where PD1 is predominantly found [97]. Thus, we speculate here that at least some of the age-related differences we have observed in our analysis might be because of differences in the TME between different patient groups (Figure 3). In this regard, it will be important to determine if EBV regulates the TME differently in these groups. We already know that EBV is a major regulator of the TME; effects that are mediated by LMP1 [98], LMP2A, and EBNA1 [99,100]. Conversely, the TME can also regulate virus gene expression in tumour cells [101,102].

In summary, a re-evaluation of the published literature presented here shows that in older adults and the elderly, an EBV-positive status is associated with poorer outcomes and that EBV-targeted therapies could be particularly valuable in this group of patients. Our literature review combined with a new analysis of a clinical trial cohort of children and adolescents with cHL suggest that while EBV-targeted therapies could also be useful in children, measuring EBV status might also be helpful in stratifying patients in future clinical trials.

## 5. Conclusions

Our literature review of the effects of EBV on outcomes for cHL patients combined with our own analysis of a cohort of paediatric and adolescent patients has shown important age-related effects. Currently, treatment for cHL patients is not stratified by EBV status, but this should be considered in future studies. For example, EBV status could be evaluated with the aim of reducing the harmful effects of harsh chemotherapeutic regimens used to treat cHL patients, particularly in childhood where there is a high risk of secondary malignancy and other complications. There is also a need to explore new opportunities to target EBV specifically, for example, with EBNA1 inhibitors or immunotherapies that target the virus. This could be particularly relevant for older adult/elderly patients where outcomes for EBV-positive patients are especially poor.

## Figures and Tables

**Table 1:** Summary of the impact of EBV on clinical outcome from published literature. Nodular lymphocyte-predominant Hodgkin lymphoma (NLPHL) cases were included in some studies. POS: positive effect of EBV on clinical outcome; NEG: negative effect of EBV on clinical outcome. “no” means no significant effect of EBV on clinical outcome. “No entry” means that the effect on clinical outcome was not studied in that group. * This was a meta-analysis that did not take age into account.

Study	Population	No. of Patients	NLPHL Included	Age (Years)	Effect (No Age Split)	Children/Adolescents	Young Adults	Older Adults
Claviez (2005) [63]	Multinational	842	yes	2–20		NEG		
Koh (2018) [64]	South Korea	135	no	<15		NEG		
Dinand (2007) [60]	India	118	yes	<15		no		
Aktas (2007) [61]	Turkey	63	no	Paediatric patients		no		
Chabay (2008) [62]	Brazil, Argentina	176	yes	0–18		no		
Engel (2000) [58]	South Africa	36	no	≤14		POS		
Keegan (2005) [46]	USA	922	no	up to 96		POS	no	NEG
Barros (2010) [59]	Brazil	104	no	up to 18		POS		
Koh (2012) [53]	S Korea	159	yes	4–77	NEG		NEG	
Jarrett (2005) [47]	UK	437	no	16–74	NEG		no	NEG
Clarke (2001) [48]	USA	311	yes	19–79			no	NEG
Kwon (2006) [54]	Korea	56	yes	6–77	NEG		POS	
Glavina-Durdov (2001) [55]	Croatia	100	yes	13–84	no		POS	
Murray (1999) [56]	UK	190	yes	22–49			POS	
Flavell (2003) [57]	UK	273	yes	≥15	no		POS	
Stark (2002) [49]	UK	102	yes	≥60				NEG
Diepstra (2009) [50]	Netherlands	412	no	7–91	no			NEG
Wang (2021) [51]	China	134	yes	5–74	no			NEG
Enblad (1999) [65]	Sweden	117	yes	11–87	NEG			
Proctor (2002) [52]	UK	94	no	>60				no
Herling(2003) [66]	USA, Italy, Greece	303	no	adults	no			
Axdorph (1999) [67]	Sweden	95	no	14–77	no			
Enblad (1997) [68]	Sweden	107	yes	6–87	no			
Keresztes (2006) [69]	Hungary	109	no	>61	no			
Krugmann (2003) [70]	Austria	119	no	14–83	POS			
Naresh (2000) [71]	India	110	no	4–61	POS			
Morente (1997) [72]	Spain	140	yes	5–83	POS			
Montalban (2000) [73]	Spain	110	yes	NK	POS			
Trimeche (2007) [74]	Belgium	111	no	8–88	NEG			
Quijano (2004) [75]	Columbia	67	no	NK	POS			
Myriam (2017) [76]	Tunisia	131	no	4–83	NEG			
Santisteban-Espejo (2021) [77]	Spain	88	no	19–82	NEG			
Elsayed (2014) [78]	Japan	389	no	4–89	NEG			
Souza (2010) [79]	Brazil	97	no	>18	no			
Cheriyalinkal Parambil (2020) [80]	India	189	no	≥15	POS			
Vestlev (1992) [81]	Denmark	66	no	12.8–60.5	no			
Armstrong (1994) [82]	UK	59	yes	NK	no			
Levy (2000) [83]	Israel	134	yes	4–50+	NEG			
Vassalo (2003) [84]	Brazil	78	no	>15	POS			
Lee (2014) [85]	various	*	NA	NA	no			

**Table 2:** Comparison of clinical trial patients included or not included in the EBV study. Interquartile range (IQR).

	In EBV Study (*n* = 189)	Not in EBV Study (*n* = 198)	*p*-Value
Age at diagnosis			0.9
Median, IQR	13.32 (10.25–14.86)	13.20 (10.00–14.69)	
Gender			0.9
Male	119 (65.0%)	116 (58.5%)	
Female	64 (35.0%)	82 (41.5%)	
Subtype			0.8
Nodular sclerosing	126 (75.9%)	129 (65.1%)	
Mixed cellularity	26 (15.6%)	39 (19.6%)	
Other/unknown	14 (8.4%)	30 (15.1%)	
Stage			0.2
I	23 (12.5%)	31 (15.6%)	
II	77 (44.8%)	103 (52.0%)	
III	38 (22.4%)	32 (16.1%)	
IV	37 (20.2%)	32 (16.1%)	
Symptoms			0.9
A	98 (59.0%)	114 (57.5%)	
B	68 (41.0%)	84 (42.4%)	

**Table 3:** Clinicopathological variables according to EBV status. Stage, subtype and symptoms were defined following review. Interquartile range (IQR).

	EBV+ (*n* = 62)	EBV− (*n* = 104)	*p*-Value
**Age at diagnosis**			<0.001
Median, IQR	10.0 (7.1–13.8)	14.2 (12.2–15.3)	
**Gender**			0.067
Male	44 (71.0%)	59 (56.7%)	
Female	18 (29.0%)	45 (43.3%)	
**Subtype**			0.005
Nodular sclerosing	41 (66.1%)	85 (81.7%)	
Mixed cellularity	17 (27.4%)	9 (8.7%)	
Other/unknown	4 (6.5%)	10 (9.6%)	
**Stage**			0.027
I	8 (12.9%)	5 (4.8%)	
II	29 (46.8%)	48 (46.2%)	
III	18 (29.0%)	22 (21.2%)	
IV	7 (11.3%)	29 (27.9%)	
**Symptoms**			0.897
A	37 (59.7%)	61 (58.7%)	
B	25 (40.3%)	43 (41.3%)	

## Data Availability

The data presented in this study are available in this article and supplementary material.

## Supplementary Materials

The following supporting information can be downloaded at: https://www.mdpi.com/article/10.3390/cancers14174297/s1, Table S1: Supplementary Table S1, rawData.xls.
